# Cytokines induced killer cells produced in good manufacturing practices conditions: identification of the most advantageous and safest expansion method in terms of viability, cellular growth and identity

**DOI:** 10.1186/s12967-018-1613-5

**Published:** 2018-08-29

**Authors:** Castiglia Sara, Adamini Aloe, Rustichelli Deborah, Castello Laura, Mareschi Katia, Pinnetta Giuseppe, Leone Marco, Mandese Alessandra, Ferrero Ivana, Mesiano Giulia, Fagioli Franca

**Affiliations:** 1 City of Health and Science Hospital of Turin, Pediatric Oncoematology, Regina Margherita Children’s Hospital, Piazza Polonia 94, 10126 Turin, Italy; 2 Department of Public Health and Pediatrics, University of Turin, 10126 Turin, Italy; 3 Medical Oncology and Experimental Cellular Therapy, Department of Oncology, Candiolo Cancer Institute IRCCS, Strada Provinciale, 142, 10060 Candiolo, Turin, Italy

**Keywords:** Validation, GMP, Clinical translation, Cellular therapy

## Abstract

**Background:**

Cytokine-induced killer (CIK) cells are a very promising cell population raising growing interest in the field of cellular antitumor therapy. The aim of our study was to validate the most advantageous expansion method for this advanced therapy medicinal product (ATMP) and to translate it from preclinical field to good manufacturing practices (GMP). GMP ensures that ATMP are consistently produced and controlled to the quality standards required to their intended use. For this reason, the use of the xenogenic sera tended to be minimized by GMP for their high variability and the associated risk of transmitting infectious agents.

**Results:**

We decided to replace Fetal Bovine Serum (FBS), largely used as medium supplement for CIKs expansion, with other culture media. Firstly, Human Serum (HS) and Human Pool Plasma (HPP) were tested as medium supplements giving not compliant results to acceptance criteria, established for CIKs, probably for the great batch to batch variability. Consequently, we decided to test three different serum free expansion media: X-VIVO 15, (largely used by other groups) and Tex Macs and Cell Genix GMP SCGM: two GMP manufactured media. We performed a validation consisting in three run-sand even if the small number of experiments didn’t permit us to obtained statistical results we demonstrated that both X-VIVO 15 and Tex Macs fulfilled the quality standards in terms of cellular growth, viability and identity while Cell Genix GMP SCGM resulted not compliant as it caused some technical problems such as high mortality.

**Conclusion:**

In conclusion, these preclinical validation data lay the bases for a GMP-compliant process to improve the CIKs expansion method.

## Background

Cytokine-induced killer (CIK) cells are a very promising cell population which raise growing interest in the field of cellular antitumor therapy mainly due to their easy expansion method.

The clinical translation of adoptive immunotherapy with CIK cells as treatment for patients with solid tumors is currently the object of clinical trials and their number has increased in recent years [1]. They can be classically expanded starting from peripheral blood mononuclear cells (PBMCs) [2, 3] but may also be generated from bone marrow (BM) or umbilical cord blood precursors [4]. Sangiolo et al. performed preliminary study in which CIKs were expanded in RPMI + 10% Fetal Bovine Serum (FBS) to evaluate their effectiveness for antitumor activity against autologous bone sarcomas [5]. CIK cells with CD3+CD56+ immunophenotype have cytotoxic effects similar to those previously obtained by other groups [3, 6, 7], as they efficiently killed sarcoma cells. Therefore, regulatory guidelines for Good Manufacturing Practices (GMP) production, aimed to minimize FBS, used in most ex vivo expansion protocols as medium supplement. This is due to the xenogenic sera high batch-to-batch variability and to the associated risk of transmitting infectious agents [8].

On these bases, our aim was translating the expansion method from preclinical to GMP field. As reported in Directive 1394/2007, in which “the principles and guidelines of GMP in respect of medicinal products for human use and investigational medicinal products for human use” are defined, the safest and the most advantageous CIKs expansion method that will be validated [9, 10]. GMP ensures that ATMP are consistently produced and controlled to the quality standards required to their intended use, from the collection and manipulation of raw materials to the processing of intermediate products, quality controls, storage, labelling and packaging, and release [11]. Moreover, according to GMP guidelines imposing to provide acceptance criteria for each pharmaceutical ATMP, and on the bases of our preclinical data, we have established that CIKs were compliant when viability was > 80%, CD3+ > 80% ± 10%, CD3+CD56+ ≥ 15% [5, 7].

## Methods

### Aim, study design and setting of the study

The aim of the study was to validate a GMP method for CIKs production as ATMP for treatment of human sarcomas. In order to replace FBS, commonly used for CIKs expansion, we decided to test Human Serum (HS) and Human Pool Plasma (HPP) as supplement medium. FBS was used as a control condition as our preclinical data were obtained culturing CIKs with RPMI + 10% FBS [5].

Each expansion run started from peripheral blood (PB) and ended with the evaluation of cellular viability, growth in terms of fold increase, identity and cytotoxicity.

As GMP guidelines recommend, blood has to be kept in quarantine in a blood bank refrigerator until the results of virology and sterility tests are provided. We first tested PB stability at 24 and 48 h from donor’s harvesting. From the comparative study, we observed no differences in terms of viability, cellular growth and identity between CIKs that expanded from fresh PB and those processed at 24 and 48 h from collection (data not shown). We could therefore exert that PB is stable up to 48 h from harvesting and this allows to wait for the virologic test results before starting the expansion process.

HS is a commercial serum while HPP is produced by the Center for Production and Validation of Hemocomponents (C.P.V.H.), City of Health and Science Hospital of Turin. From literature emerged that HPP was already used instead of FBS or HS for CIKs GMP compliant expansion and that the presence of fibrinogen in HPP did not interfere with the lymphocyte expansion [12].

HPP was also subjected to Pathogen Inactivation (PI) technique by riboflavin (45–85 μM) and UV light (265–370 nm). Riboflavin, commonly used to treat platelets or plasma (MIRASOL™ Pathogen Reduction Technology System, Caridian BCT Biotechnologies, Lakewood, Colorado, USA), is a naturally occurring vitamin (vitamin B2) used as a photosensitizer in combination with light. It provides energy to inactivate living micro-organisms [13]. This process could guarantee the safety of culture medium for CIKs expansion in a GMP setting as it might obviate virus transmission problems [14]. In order to reduce donor specific batches variability, HPP was obtained through centrifugation of whole blood of multiple donors and contains a variety of valuable organic and inorganic elements. For these reasons, HPP seemed a good alternative to FBS for CIKs expansion.

Unfortunately, we obtained contradictory results (data not shown). We demonstrated that HS was not suitable for our aim as it drastically compromised the quality of CIKs expansion in terms of cellular viability, growth and identity. On the contrary, the RPMI + 10% HPP expanded CIKs had grown up more than those in HS with a fold increase similar to those of RPMI + 10% FBS expanded CIKs. However, this trend was not maintained in the following expansion runs and the immunophenotype was not always compliant to the acceptance criteria demonstrating the presence of a great variability between HPP batches. This was a bad characteristic which compromised the choice of this supplement for the CIKs expansions. Moreover, the need to freeze and thaw HPP for its storage could bring to radical oxygen species formation [15] which are toxic causing the cells viability and growth decrement.

For all these reasons we tried to find an alternative expansion medium less variable but as efficient as RPMI + 10% FBS. We decided to test three different serum free expansion media: X-VIVO 15, largely used by other groups [16] and two GMP manufactured media: Tex Macs and Cell Genix GMP SCGM. Viability, cellular growth in terms of fold increase, immunophenotype and cytotoxicity were evaluated and compared at the end of each expansion run.

### Peripheral blood collection and PBMCs isolation

The Center for Production and Validation of Hemocomponents (C.P.V.H.) of the City of Health and Science Hospital of Turin provided the whole Peripheral blood from healthy donors. For ethical reasons, we received, after informed consent, only the PB which were not conform for donation purposes and so considered a waste material to be eliminated (examples: donations interrupted for technical problems in the collection with a volume lower than the reference values for the acceptance of transfusion material).

**Fig. 1:**
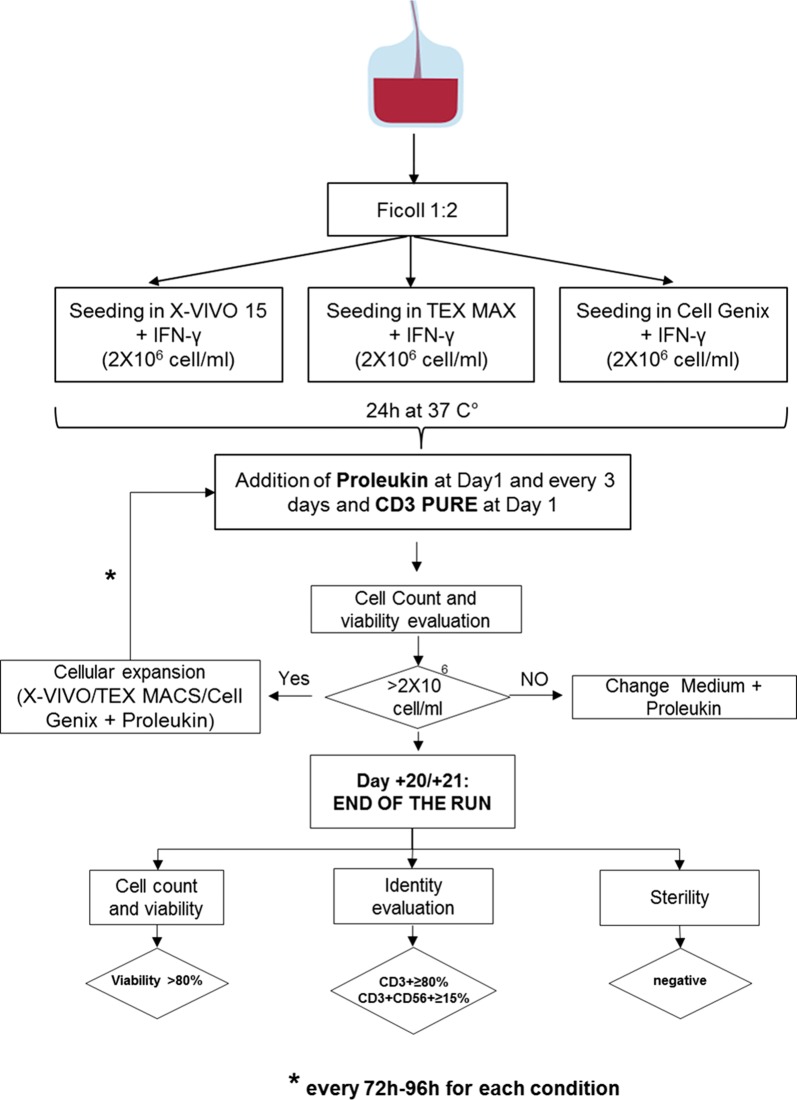
Methodological flow chart. Graphical Illustration of the experimental steps of CIKs expansion method in the three culturing conditions: X-VIVO 15, Tex Macs and Cell Genix GMP SCGM

The raw material was collected and validated by C.P.V.H., which verified the compliance of all virology tests carried out on the collected PB. Blood was stored in the blood bank refrigerator (+ 4 °C) and kept in quarantine until the results of virology tests were provided. After about 24 h from collection, the PB was processed for CIKs production. The study design is illustrated in Fig. 1.

PBMCs were separated by density gradient centrifugation using HISTOPAQUE-1077 (Sigma-Aldrich St. Louis, US) and subjected to washing cycles in Phosphate buffered saline (PBS) (EuroClone, Pero, MI, Italy) and finally re-suspended in the culture medium at the cellular concentration of 2 × 10^6^ cells/ml.

### CIKs expansion

We compared three different serum-free culture media in order to obtain the best expansion condition: X-VIVO 15 (Lonza, Allendale, NJ, US), largely used in the clinical grade lymphocytes expansion [16] and other two culture media generated in GMP conditions: Cell Genix GMP SCGM (CellGenix, Freiburg, Germany) and Tex Macs (Miltenyi Biotec, Bergisch Gladbach, Germany). Briefly, 30 × 10^6^ of cells were seeded in 15 ml of culture medium. We cultured the CIKs in T75 flasks (Corning Incorporated, NY, USA). They were not a treated plastic type and they were endowed of vented cap. They were maintained in a vertical position in order to ensure a better oxygenation of cellular suspension, to reduce the culturing medium distribution surface and to have an appropriate culturing medium volume in order to re-suspend homogeneously the cells.

At Day 0, 1000 U/ml IFN-γ was added (Boehringer Ingelheim, Vienna, Austria). At Day 1, 50 ng/ml CD3 Pure, which is IgG anti-CD3 (OKT-3) (170-076-124, Miltenyi Biotec, Bergisch Gladbach, Germany), and 300 IU/ml of Proleukin which is IL-2 cytokines (Novartis, Origgio, VA, Italy) were added [4]. Fresh medium and Proleukin (300 U/ml) were added weekly (every 3 days) during culture, and the cell concentration was maintained at 1-1.5 × 10^6^ cells [17]. The fold increase of CIKs was calculated as follow: (%CD3+ × total cells counted at Day 20–21)/(%CD3+ × seeded cells at Day 0).

After 20–21 days, CIK cells culture was stopped and the quality controls were performed (cell count and viability, immunophenotype and sterility).

### Cell count and viability evaluation

CIK cells were counted at optical microscope, using Burker chamber as indicated in the European Pharmacopoeia (Chapter 2.7.29) [18]. The best counting condition in order to obtain a measurement as reliable as possible was between 80 and 150 cells in 25 squares of Burker chamber. The cellular viability was evaluated with Trypan Blue Method (Sigma-Aldrich, St. Louis, US) [18].

### Flow cytometry

Flow cytometry was performed according to European Pharmacopoeia (Chapter 2.7.24) using Beckman Coulter NAVIOS (Beckman Coulter, Brea, CA, US) [19]. At Day 0, before PBMCs seeding, basal flow cytometry was performed in order to evaluate the lymphocyte subpopulations. About 0.5–1 × 10^6^ cells were incubated for 20 min, at 4 °C in the dark with respectively CYTO-STAT TetraCHROME CD45-FITC, CD56-RD1, CD19 ECD, CD3-PC5 (Beckman Coulter, Brea, CA, US) for B lymphocytes and CYTO-STAT TetraCHROME CD45-FITC, CD4-RD1, CD8-ECD, CD3-PC5 (Beckman Coulter, Brea, CA, US) for T lymphocytes. Cells were washed and re-suspended in 300 µl of PBS. After 20–21 days of expansion flow cytometry was performed to evaluate the CIKs identity. Briefly, 0.5–1 × 10^6^ cells were incubated as previously described with CD3-FITC, CD56-PE, CD45 KO (Beckman Coulter, Brea, CA, US). As negative control, 0.5–1 × 10^6^ cells were incubated without antibody. For data analysis, we designed the physical gate as [A]. From this gate, we obtained the CD3+ and the CD56+ cell population. In the dot plot CD3xCD56 we gated the CIK double positive CD3+CD56+ cell population. The tail is representative of the primitive CD3+CD56+. These cells were the cellular therapy product as it was in compliance with the identity acceptance criteria.

### CIKs cytotoxicity

CIKs tumor-killing ability was assessed against target primary tumor cells obtained from surgical biopsies of gastro-intestinal stromal tumor (GIST).

CIK cells were co-cultured at progressively decreasing effector:target (E:T) ratios, 40:1, 20:1, 10:1, 5:1, 2.5:1, 1:1, 1:2, and 1:4 for 72 h in 200 μl of medium with Proleukin at a concentration of 300 U/ml at 37 °C 5% CO_2_. A confirmatory method was tested in parallel to determine the number of viable, metabolically active, target cells in culture, based on the quantification of ATP present (CellTiter-Glo Luminescent Cell Viability Assay, Promega Italia s.r.l.). Tumor cells, in the absence of CIK cells, were used as a control to assess spontaneous mortality. The percentage of tumor-specific lysis for each E:T ratio was calculated as experimental − spontaneous mortality/100 − spontaneous mortality × 100 [5].

### Sterility

Sterility test was performed on cellular supernatants at the end of CIKs expansion. Briefly, 10 ml and 4 ml of supernatant respectively for anaerobic and aerobic microorganisms and fungal/yeasts species, were inoculated within the Bact-ALERT^®^ FN and Bact-ALERT^®^ PF (Biomérieux, INC. Durham, NC). The sample was then analyzed by the Laboratory of Bacteriology and Virology—Pediatric Clinical Pathology of City of Health and Science of Turin. The results were available after 7–8 days of incubation.

### Statistical analysis

Cell growth, viability, immunophenotype and cytotoxicity assay data were analyzed with the GraphPad program (version Prism 5). The data were expressed as mean ± standard deviation. The statistical tests were chosen on the basis of the number of samples and the distribution of the data. The latter was evaluated by Shapiro–Wilk Test, which is considered the best for the normality evaluation for a small number of samples. Paired *T* test was performed when the distribution was normal, on the contrary, when there was not a Gaussian distribution, Wilcoxon Rank Test was used. Finally, Bonferroni’s multiple comparison Test was conducted to compare the mean values on each data row, which represent a condition, with the control mean data row.

## Results

### Comparison between culture expansion media

We compared X-VIVO 15 with other two serum-free culture media: Cell Genix GMP SCGM and Tex Macs, both manufactured in GMP conditions.

Three validation runs were performed and viability, cellular growth, identity and cytotoxicity were evaluated at Day 20–21 of CIKs expansion and compared in the three culture conditions.

**Fig. 2:**
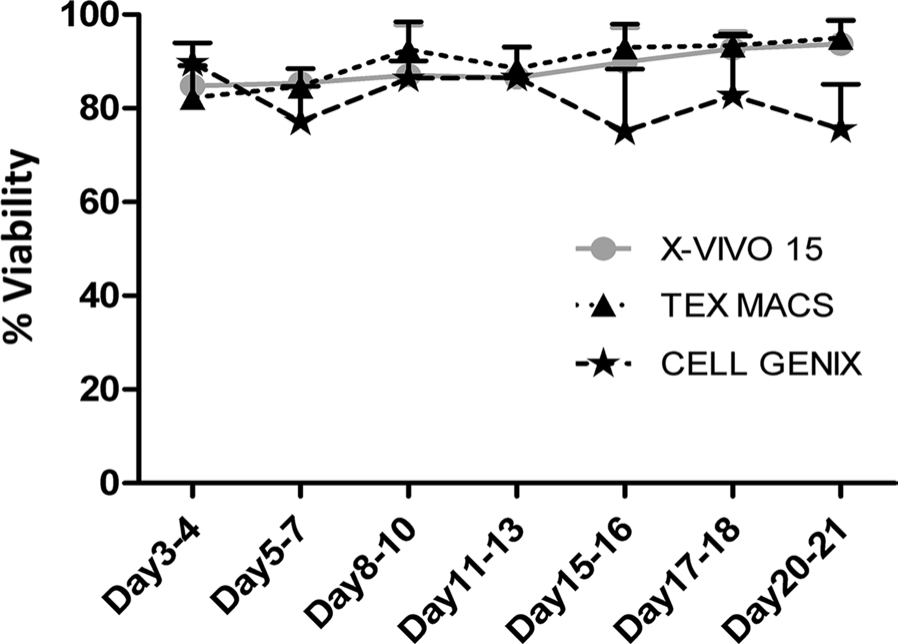
Viability study between X-VIVO 15, Tex Macs and Cell Genix GMP SCGM. All data were analyzed with the Paired-T statistical Test and represented as mean ± St.Dev (N = 3)

Viability data of each culture condition were analyzed by the Paired T-test that did not highlight any statistical differences between studied groups. Nevertheless, the CIKs viability recorded at Day 21 of expansion in Cell Genix GMP SCGM was not compliant in two of the three runs (viability < 80%) (data not shown). The results are shown in Fig. 2.

**Fig. 3:**
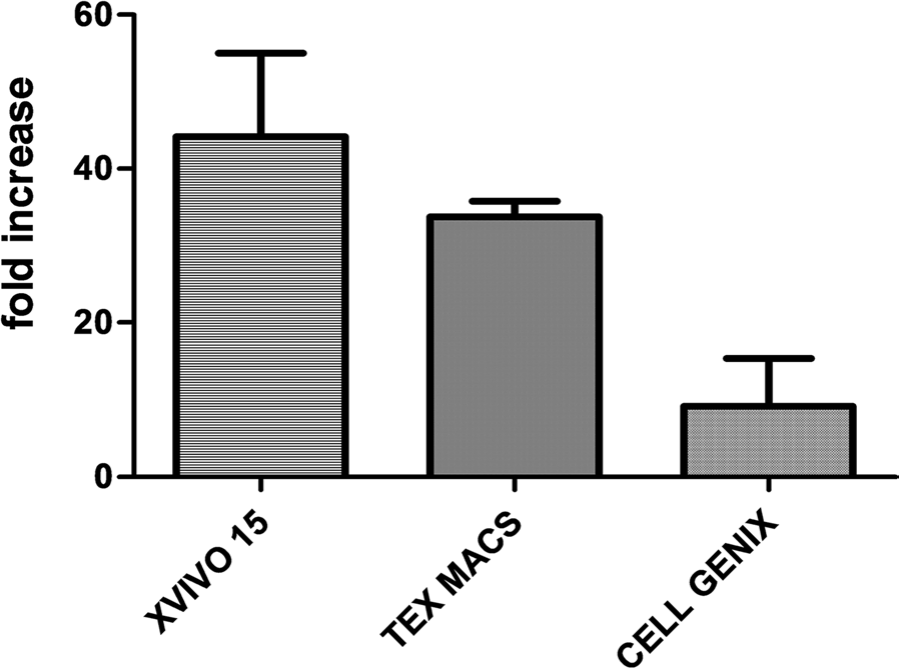
Statistical analysis of cellular growth expressed as fold increase. Data were analyzed with the Paired-T statistical Test and represented as mean ± St.Dev (N = 3)

Cellular growth was expressed as fold increase of CD3+ from Day 0 to Day 20–21. As shown in Fig. 3, CIKs expanded in Cell Genix GMP SCGM, grew less than both X-VIVO 15 and Tex Macs (X-VIVO 15: 47.11 ± 31.79; Tex Macs: 34.44 ± 6.42; Cell Genix GMP SCGM: 9.17 ± 10.80).

**Fig. 4:**
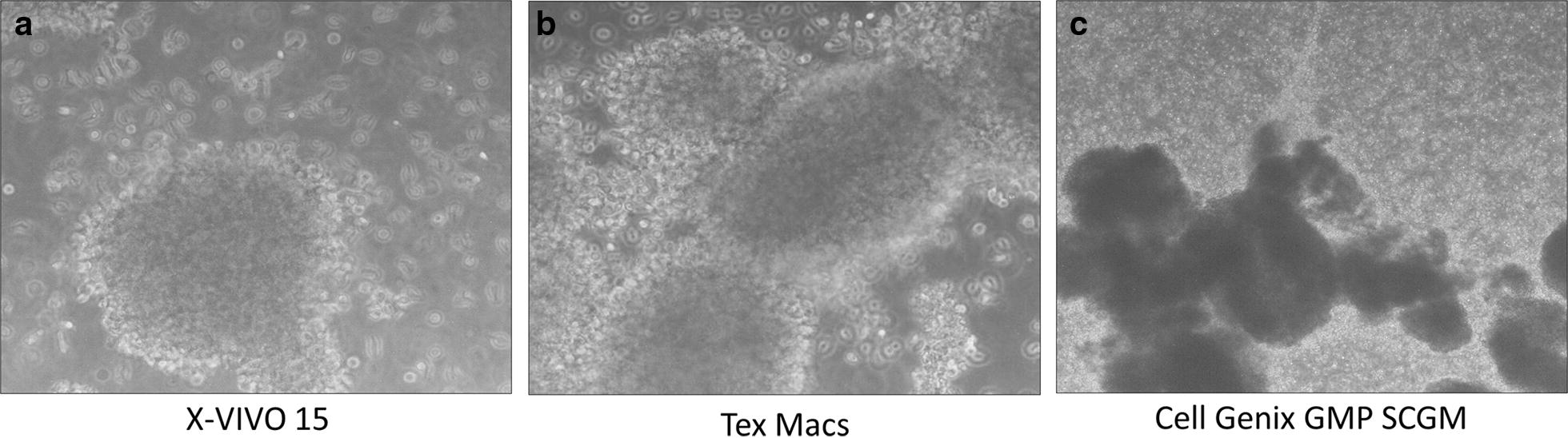
Representative Morphological image of CIKs’ clusters/aggregates in the three culturing conditions: X-VIVO 15 (**a**), Tex Macs (**b**) and Cell Genix GMP SCGM (**c**). The analysis was performed at optic microscope 20X magnification

We observed some differences in the CIKs morphology during the expansion between the three tested conditions. In particular, CIKs expanded in Cell Genix GMP SCGM formed numerous aggregates, which were different from the classic clusters observed in X-VIVO 15 and Tex Macs cultures. These aggregates caused many difficulties in cell count procedures it resulted impossible to establish cells concentration (N° cells/ml) and viability. The abnormal agglomerates in fact were not broken by resuspension and were all positive to trypan blue staining. As shown in Fig. 4, the aggregates of Cell Genix GMP SCGM were dark and had irregular edges. In both X-VIVO 15 and Tex Max the clusters were circular and light reflective indicating the viability of the agglomerates forming cells.

**Fig. 5:**
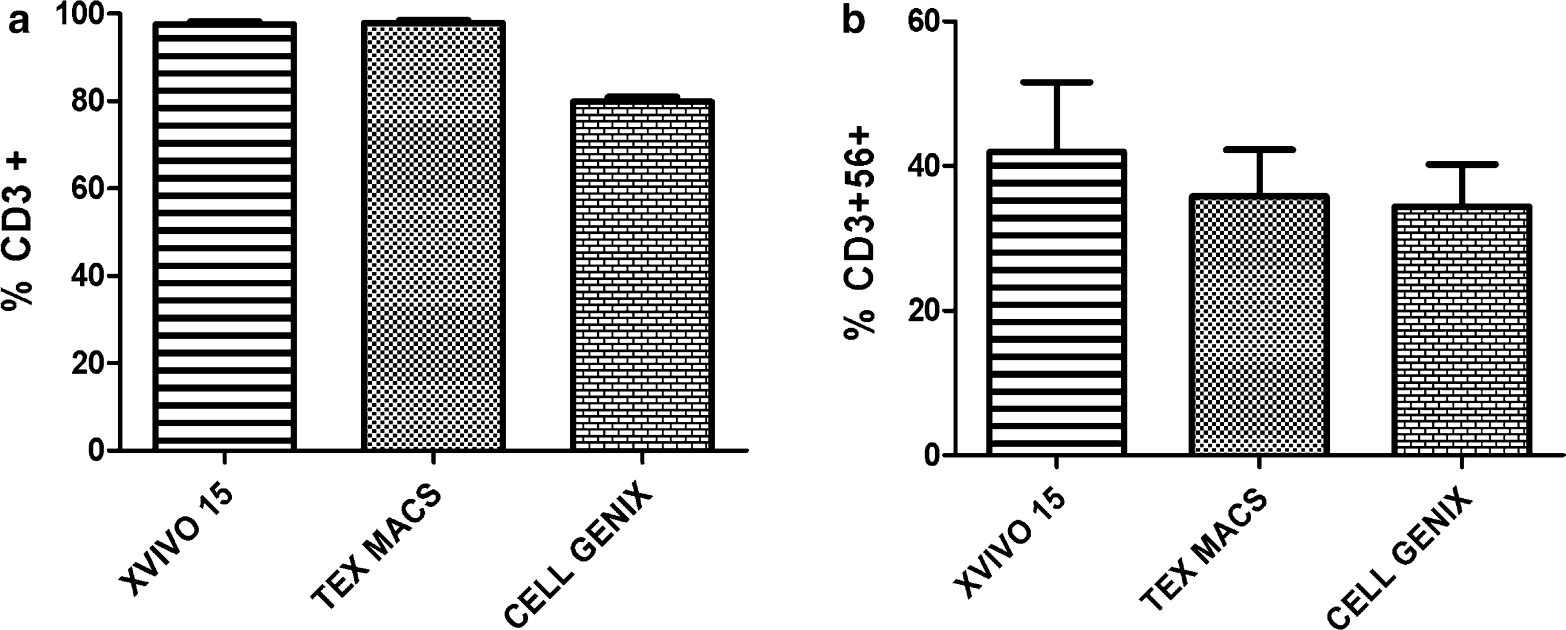
Percentage of CD3+ (**a**) and CD3+CD56+ (**b**) in the three studied conditions. Data were analyzed with the Paired-T statistical Test and represented as mean ± St.Dev (N = 3)

Moreover, the X-VIVO 15 and Tex Macs conditions had higher CD3+ levels than those found in the Cell Genix GMP SCGM condition. (X-VIVO 15: 97.56 ± 1.15; Tex Macs: 97.84 ± 1.27; Cell Genix GMP SCGM: 79.85 ± 1.94) (Fig. 5a). The Cell Genix GMP SCGM condition was not compliant to the release criteria in term of CD3+ % in one of the three validation runs (CD3+ % = 77.62). Similarly, the percentage of CD3+56+ cells was higher in the X-VIVO 15 and Tex Macs conditions compared to the Cell Genix one. Although the percentage of CD3+56+ was higher in X-VIVO 15 than in Tex Macs, there were no statistically differences (X-VIVO 15: 41.91 ± 16.76; Tex Macs: 35.76 ± 11.16; Cell Genix GMP SCGM: 34.36 ± 10.18) (Fig. 5b).

**Fig. 6:**
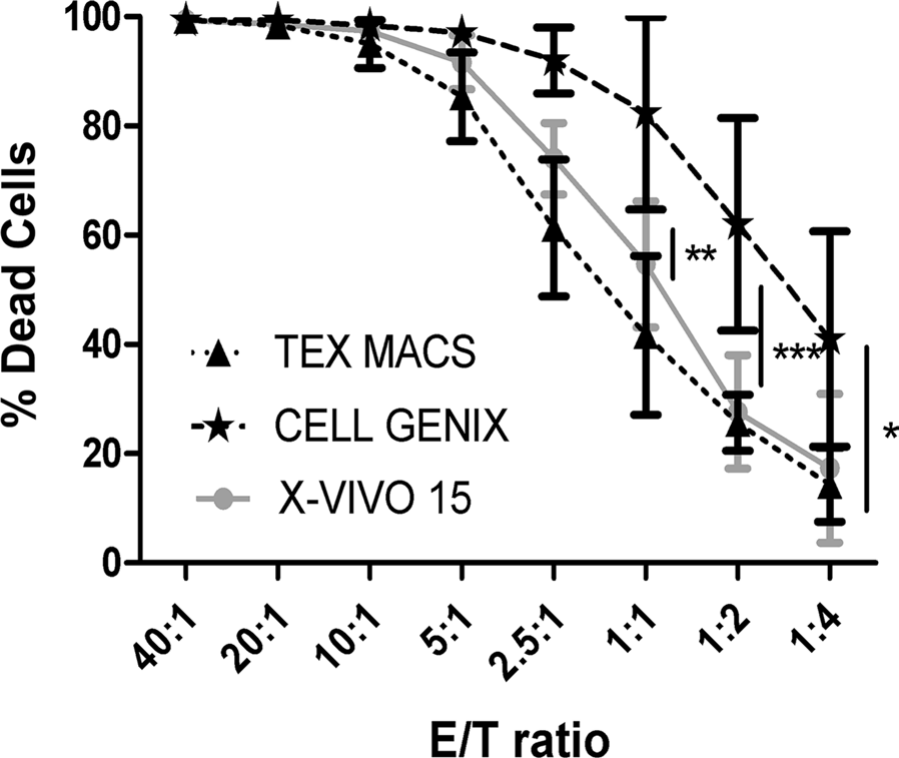
Graphic representation of the percentages of death cells (Target) at each E/T ratios. The comparison was between X-VIVO 15 *vs* Tex Macs vs Cell Genix GMP SCGM. Data were expressed as mean ± St. Deviation. The statistic test performed was Two Way RM ANOVA with Bonferroni Post Test; (N = 3); *p < 0.05. **p < 0.01, ***p < 0.001

Finally, Cell Genix GMP SCGM CIKs showed a statistically higher cytotoxic action than both X-VIVO 15 and Tex Macs CIKs. (i.e. E/T ratio 1:1 → X-VIVO 15: 55 ± 11.61; Tex Macs: 42 ± 14.86; Cell Genix GMP SCGM: 82 ± 17.47). The difference is statistically significant (p < 0.01) also between X-VIVO 15 vs Tex Macs (Fig. 6).

### Sterility

Sterility test was performed at the end of each validation run of expansion. All the runs resulted compliant, as bacteriology report was always negative for the presence of Gram + and Gram- Bacteria, Fungal/yeasts.

## Discussion

CIK cells are a heterogeneous subset of polyclonal T cells with the phenotype CD3+CD56+ and functional properties of NK cells. They rise from CD3+ T cell precursors and during the expansion acquire the expression of CD56+. Furthermore, CIK cells CD3+CD56+ are endowed with a potent MHC-unrestricted cytotoxicity against tumor target. Their antitumor activity is due to their cytotoxic action mediated by the perforins and by the interaction Fas–Fas ligand [2]. They were firstly clinically tested against onco-haematology malignancies [4, 16] and then against solid tumors [5, 7]. For this reason, they have emerged as important ATMP for the therapy of a lot of malignancies such sarcomas [5]. Their development has to follow a specific regulatory framework which is applied to traditional pharmaceutical drugs. On these basis, CIKs production is governed in Europe by the Directive 2001/83/EC, amended by Regulation 1394/2007 [9, 10].

Our aim was to extend the CIKs expansion method from preclinical field to GMP. We replaced FBS, in accordance to GMP guidelines that limit the xenogenic serum for human use. We tried to use as supplement medium HS and HPP but the obtained results demonstrated a great variability in HPP batches. On the other hand, HS was not a good alternative to FBS.

For these reasons we decided to test serum free medium by comparing X-VIVO 15, commonly used in CIKs expansion [4], Tex Macs and Cell Genix GMP SCGM, both produced in GMP conditions.

The results were not significantly different as the number of experiments was too low and there was a great variability due to the raw CIKs cultures started materials. In this validation in fact, three runs were sufficient to underline methodological criticisms and to identify the best culturing condition, but not to obtain statistically differences. Both X-VIVO 15 and Tex Macs proved to be valid for CIKs expansion in terms of cell growth, viability and cell identity. In fact, as previously described X-VIVO 15 and Tex Macs expanded CIKs were more satisfactory in terms of cellular viability and identity than those expanded with Cell Genix (Figs. 2, 3, 5). Moreover, Cell Genix GMP SCGM could promote aggregates formation which caused several technical problems in terms of cellular resuspension during cell count (Fig. 4). The microscopic observation led us to demonstrate that they were not active CIKs clusters but dead cells aggregates as they were positive to trypan blue staining. This was a very critical aspect as it compromised the cellular viability and growth evaluation. An accurate cell count represents a crucial point for a correct concentration cell seeding (1–1.5 × 10^6^/ml). The CIKs potency against tumor cells was measured by citotoxicity test: in general CIKs cells recognize tumor targets and kill them without prior exposure or priming. The NKG2D molecule expressed on the membrane of CIK plays a pivotal role in their mechanism of action as it recognizes and interacts with stress inducible molecules MHC-unrestricted expressed only by tumor cells [17].

**Fig. 7:**
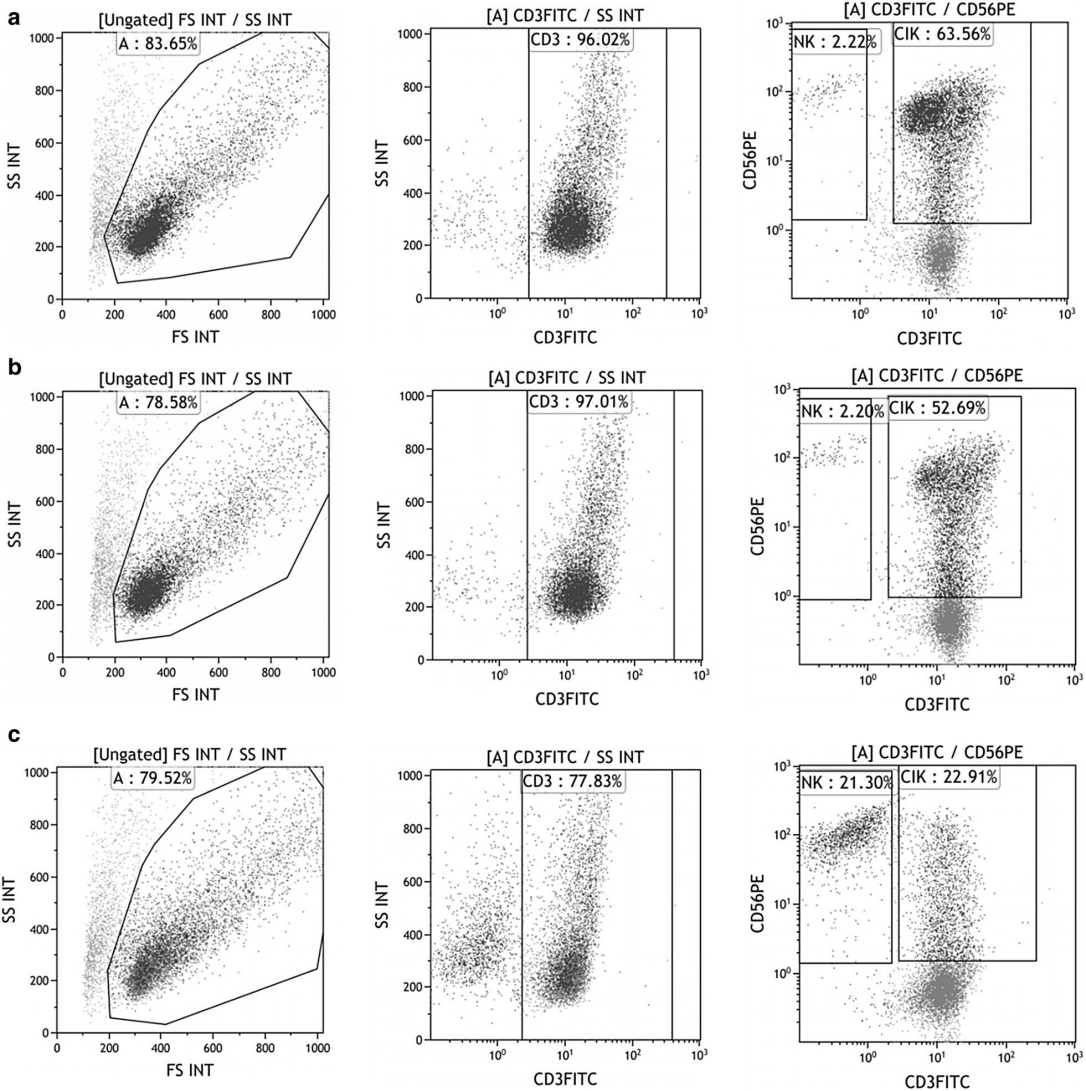
Representative of CIKs immunophenotype. The comparison was between X-VIVO 15 (**a**) vs Tex Macs (**b**) vs Cell Genix GMP SCGM (**c**)

By CIKs cytotoxicity test emerged that Cell Genix GMP SCGM was the most cytotoxic condition, statistically different from the other two. Then, X-VIVO 15 cytotoxicity was statistically higher than Macs (Fig. 6). As Cell Genix GMP SCGM is a culture medium specific for NK expansion it was able to expand a significant amount of CD3−CD56+ cells if compared to X-VIVO 15 and Tex Macs, (Fig. 7c). This confirmed that a higher amount of NK cells make cellular suspension more cytotoxic [20]. This phenomenon has already been observed in CIKs expanded with RPMI + 10% HPP (data not shown). This would explain also the least CD3+ amount in Cell Genix GMP SCGM (Fig. 7c) compared to X-VIVO 15 and Tex Macs cultures (Fig. 7a, b).

Greater cytotoxicity certainly was a good measure of ATMP effectiveness against the tumor cell targets. Anyway, as GMP CIKs expansion was our main purpose, we could not neglect that Cell Genix was not a good medium. In fact, two of three validation runs were not compliant to release criteria in terms of viability (> 80%) and CD3+ expression (> 80%)and the presence of dead cells clusters, could negatively affect the entire expansion process and the ATMP quality. On the other hand, both X-VIVO 15 and Tex Macs were valid candidates for CIKs expansion.

## Conclusion

As our aim is to produce CIKs with the best GMP expansion method in Regina Margherita Children Hospital, City of health and Science of Turin Cell Factory, we tested the effectiveness of a commercial medium (X-VIVO 15) which is equivalent and less expensive than other ones produced in GMP conditions (Tex Macs and Cell Genix GMP SCGM). This preclinical validation lays the bases for a GMP-compliant CIKs expansion process.

This ATMP will be used in an autologous setting in patients affected by refractory and unresectable soft and bone sarcomas and will be administered by intravenous infusion. For this aim a phase I clinical Trial entitled “A phase I study of adoptive immunotherapy with cytokine-induced killer (CIK) cells in relapsed and non-resectable sarcomas after multimodal treatment” is now in the submission phase.

## Abbreviations

ATMP: advanced therapy medicinal products
BM: bone marrow
C.P.V.H: Center for Production and Validation of Hemocomponents
CIK: cytokine-induced killer
FBS: Fetal Bovine Serum
GIST: gastro-intestinal stromal tumor
GMP: good manufacturing practices
HPP: human pool plasma
HS: human serum
PB: peripheral blood
PBMC: peripheral blood mononuclear cells
PBS: phosphate buffered saline
PI: pathogen inactivation
