# Correction to: Cytokines induced killer cells produced in good manufacturing practices conditions: identification of the most advantageous and safest expansion method in terms of viability, cellular growth and identity

**DOI:** 10.1186/s12967-018-1656-7

**Published:** 2018-10-10

**Authors:** Sara Castiglia, Aloe Adamini, Deborah Rustichelli, Laura Castello, Katia Mareschi, Giuseppe Pinnetta, Marco Leone, Alessandra Mandese, Ivana Ferrero, Giulia Mesiano, Franca Fagioli

**Affiliations:** 1 City of Health and Science Hospital of Turin, Pediatric Oncoematology, Regina Margherita Children’s Hospital, Piazza Polonia 94, 10126 Turin, Italy; 2 Department of Public Health and Pediatrics, University of Turin, 10126 Turin, Italy; 3 Candiolo Cancer Institute, FPO-IRCCS, Turin, Italy

## Correction to: J Transl Med (2018) 16:237 10.1186/s12967-018-1613-5

Following publication of the original article [1], the authors reported that all of the authors’ names were processed incorrectly so that their given and family names were interchanged. In this Correction the correct author names are shown. The original publication of this article has been corrected.

The correct names are: Sara Castiglia, Aloe Adamini, Deborah Rustichelli, Laura Castello, Katia Mareschi, Giuseppe Pinnetta, Marco Leone, Alessandra Mandese, Ivana Ferrero, Giulia Mesiano and Franca Fagioli.
